# Cytochrome c oxidase subunit 1 gene reveals species composition and phylogenetic relationships of *Oesophagostomum* spp. infecting pigs in northeastern Brazil

**DOI:** 10.1590/S1984-29612022016

**Published:** 2022-04-01

**Authors:** Polyanna Araújo Alves Bacelar, Kerla Joeline Lima Monteiro, Deiviane Aparecida Calegar, Jéssica Pereira dos Santos, Beatriz Coronato-Nunes, Elis Regina Chaves dos Reis, Márcio Neves Bóia, Lauren Hubert Jaeger, Filipe Anibal Carvalho-Costa

**Affiliations:** 1 Laboratório de Epidemiologia e Sistemática Molecular, Instituto Oswaldo Cruz, Fundação Oswaldo Cruz – Fiocruz, Rio de Janeiro, RJ, Brasil; 2 Escritório Técnico Regional, Fundação Oswaldo Cruz – Fiocruz, Teresina, PI, Brasil; 3 Faculdade de Medicina de Petrópolis – FMP, Centro Universitário Arthur Sá Earp Neto – UNIFASE, Petrópolis, RJ, Brasil; 4 Secretaria Municipal de Saúde de Nossa Senhora de Nazaré, Nossa Senhora de Nazaré, PI, Brasil; 5 Laboratório de Biologia e Parasitologia de Mamíferos Silvestres Reservatórios, Instituto Oswaldo Cruz – Fiocruz, Fundação Oswaldo Cruz – Fiocruz, Rio de Janeiro, RJ, Brasil; 6 Departamento de Ciências Farmacêuticas, Faculdade de Farmácia, Universidade Federal de Juiz de Fora – UFJF, Juiz de Fora, MG, Brasil

**Keywords:** DNA barcode, Oesophagostomum, molecular taxonomy, pigs, Código de barras de DNA, Oesophagostomum, taxonomia molecular, suíno

## Abstract

Helminths of the genus *Oesophagostomum* cause enteric diseases and affect domestic animals such as pigs. The aim of this study was to explore the species composition and genetic diversity of *Oesophagostomum* spp. infecting pigs in close contact with humans in the state of Piauí, Brazil. Eighty-seven fecal samples were collected for parasitological tests and molecular analysis. Through microscopy, the overall positivity rate for strongyliform eggs was 81.6% among the pigs studied. Forty-two strongyliform egg samples were subjected to PCR and six *cox*1 sequences (637 bp) were identified for the genus *Oesophagostomum*. The sequences were identified as *Oesophagostomum dentatum, O. quadrispinulatum* and *O. columbianum*. In the phylogenetic tree and haplotype network, 89 sequences were separated into seven clusters, which also included reference sequences from GenBank. *Oesophagostomum dentatum* and *O. quadrispinulatum* were seen to be closely related species and formed a monophyletic group related to *O. aculeatum*. *Oesophagostomum columbianum* showed similarity with sequences from parasites infecting small ruminants and the clade was positioned closer to *O. bifurcum*. High interspecific diversity was found and intraspecific diversity varied according to the species. This was the first study to characterize *Oesophagostomum* DNA sequences obtained from pigs in Brazil.

## Resumo

Parasitos do gênero *Oesophagostomum* causam doenças entéricas e podem afetar a criação de animais, como os suínos. O objetivo deste estudo foi identificar as espécies e explorar a diversidade genética de *Oesophagostomum* spp. infectando suínos em contato próximo com humanos, no estado do Piauí, Brasil. Oitenta e sete amostras fecais foram coletadas para testes parasitológicos, análise morfométrica dos ovos e análises moleculares. A taxa geral de positividade para ovos estrongiliformes foi de 81,6%. Quarenta e duas amostras de ovos estrongiliformes foram submetidas à PCR e seis sequências *cox*1 (637 bp) foram identificadas para o gênero *Oesophagostomum*. As sequências foram identificadas como *Oesophagostomum dentatum*, *O. quadrispinulatum* e *O. columbianum*. Na árvore filogenética e na rede haplotípica, 89 sequências foram separadas em sete clusters, incluindo sequências de referência do GenBank. *Oesophagostomum dentatum* e *O. quadrispinulatum* são espécies estreitamente relacionadas e formaram um grupo monofilético com *O. aculeatum*. *Oesophagostomum columbianum* apresentou semelhança com sequências de parasitas obtidos de pequenos ruminantes e o clado foi posicionado mais próximo de *O. bifurcum*. Alta diversidade interespecífica foi encontrada e a diversidade intraespecífica variou de acordo com as espécies. Esse foi o primeiro estudo a caracterizar sequências de DNA de *Oesophagostomum* isoladas de suínos no Brasil.

## Introduction

The genus *Oesophagostomum* (family Chabertiidae, order Strongylida) includes worms ranging in length from 6.5 to 24 mm. The anterior end of the body presents a shallow oral cavity, a dilated cuticle forming a cephalic vesicle and a radiated crown present with varied structure (Taylor et al., 2010). Species within this genus are hosted by a wide diversity of mammals.

Considering only the species with the greatest impact on public health and veterinary medicine, pigs can be infected with the species *O. dentatum* and *O. quadrispinulatum* (Lin et al., 2014). *Oesophagostomum bifurcum, O. stephanostomum, O. brumpti* and *O. aculeatum* have previously been described in human and non-human primates (Glen & Brooks, 1985); and *O. columbianum, O. venulosum* and *O. asperum* in sheep and goats (Gaddam et al., 2017). Despite the first human case of ‘*O. stephanostomum*’ infection was recorded from Brazil, the actual systematic position of this worm has not been settled (Railliet & Henry, 1910; Thomas, 1910).

Oesophagostomiasis causes economic losses in pig farming, affecting both large and small producers (Li et al., 2017). Despite differences in size, Strongylida eggs are similar in morphology, which makes it difficult to identify species with parasitological examinations. Although obtaining larvae through stool cultures helps in making genus-specific diagnoses, species identification remains limited (Benavides et al., 2007).

To overcome these limitations, molecular taxonomy through DNA sequencing is widely applied to diagnoses, to enable phylogenetic reconstructions and evolutionary deductions of infectious agents (De & Bandyopadhyay, 2008). The objective of the present study is to explore the species composition and genetic diversity of Oesophagostomum infecting pigs in rural communities in northeastern Brazil.

## Material and Methods

**Figure 1:**
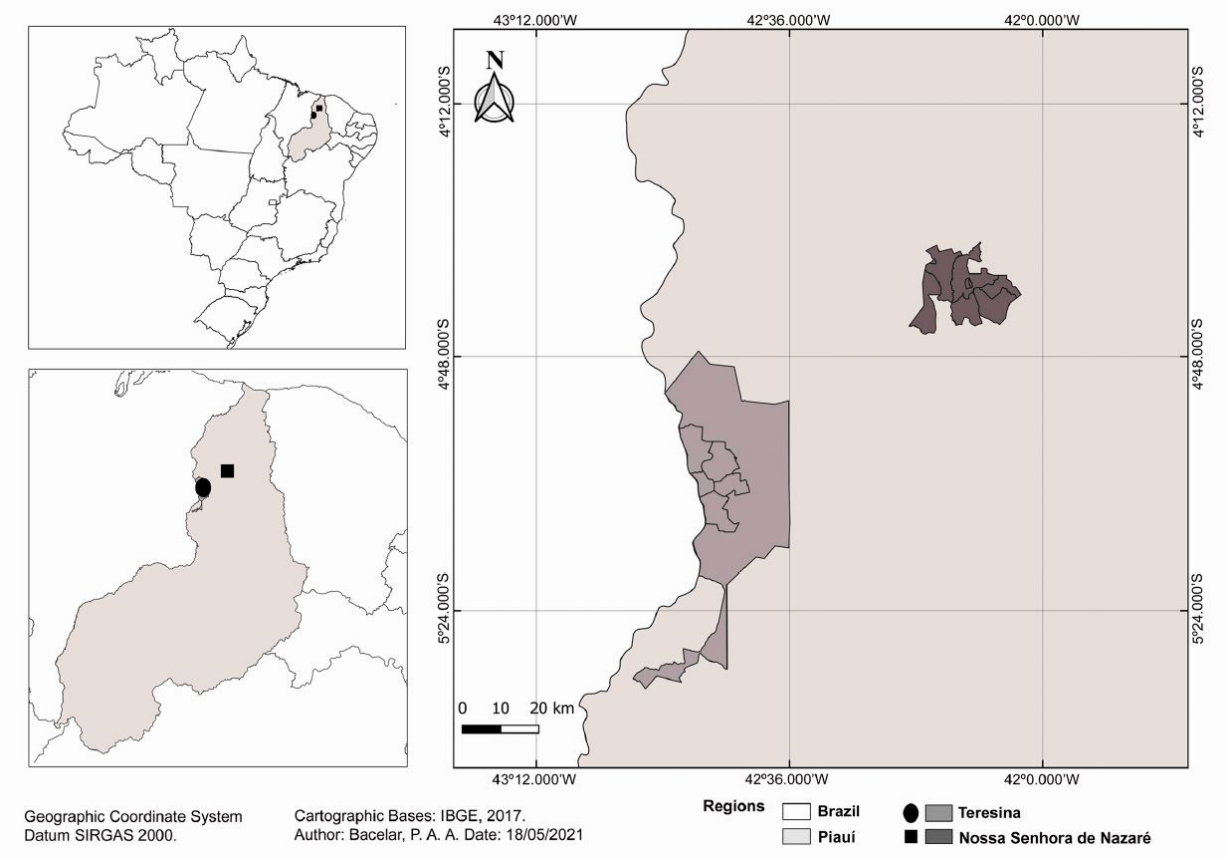
Geographical location of the study in Piauí state, northeastern Brazil.

The fieldwork was performed from 2014 to 2017 in two regions in the state of Piauí, northeastern Brazil: Nossa Senhora de Nazaré (NSN) and Teresina (TER), in periurban and rural low-resource communities (Figure 1). Fecal samples from pigs (n = 78 in NSN and 9 in TER) were collected after spontaneous defecation, on the ground. The pigs were in rudimentary shelter or in peridomestic or domestic areas during the field visits. These samples were individually stored in properly identified plastic bags, placed in a container with ice and sent to the field laboratory for parasitological examinations. The feces were processed using the Ritchie method (centrifugal sedimentation with ethyl acetate) and sucrose flotation. Samples that contained helminth eggs were then frozen until DNA extraction.

Genomic DNA was extracted from 42 fecal samples positive for strongyliform eggs using the DNeasy Blood & Tissue kit (Qiagen, Hilden, Germany), in accordance with the manufacturer's instructions. The partial cytochrome c oxidase subunit 1 (*cox*1) gene was amplified using the Platinum Taq DNA polymerase (Invitrogen, Waltham, MA, USA) with a final volume of 50 µL. A cocktail of three primer pairs that recover *cox*1 barcodes from diverse nematode lineages parasitic on vertebrates, including members of three orders and eight families was used (Prosser et al., 2013). The PCR conditions were as follows: initial denaturation at 94 ºC for 5 min, followed by 35 cycles of 94 ºC for 40 s, 55 ºC for 40 s, 72 ºC for 1 min and a final extension at 72 ºC for 5 min. The PCR products were purified using the DNA Illustra GFX PCR and gel band purification kit (GE HealthCare, Pittsburgh, USA) and were subjected to sequencing of both strands of DNA using the BigDye Terminator v. 3.1 cycle sequencing kit (Thermo Fisher Scientific, Foster City, USA) using the following primers: M13F 5'- TGT AAA ACG ACG GCC AGT – 3' (forward) and M13R 5'- CAG GAA ACA GCT ATG AC – 3' (reverse) (Messing, 1993). Capillary electrophoresis was performed using an ABI 3730 automated DNA sequencer (Applied Biosystems). Sequences showing overlapping peaks on electropherograms were cloned using the pGEM®-T Easy vector system (Promega, Madison, WI, USA) with *Escherichia coli* DH5-alpha cells in brain heart infusion broth (Sigma-Aldrich, St. Louis, MO, USA) on disposable plates. After cloning, the PCR and sequencing conditions were performed using the universal primers T7 5'-TAA TAC GAC TCA CTA TAG G - 3' (forward) and SP6 5'-GAT TTA GGT GAC ACT ATA G - 3' (reverse).

The Bioedit software v.7.0.4 (Hall, 1999) was used to edit the nucleotide sequences (401 bp). The Basic Local Alignment Search Tool (BLAST) (NCBI, 2022) was used to verify the similarity of the nucleotides with sequences of nematodes from GenBank. Orthologous sequences (n = 83) were retrieved from GenBank (Table 1) and sequences with degenerate bases were not included. The sequences obtained in the study (n = 6) were deposited in GenBank under accession numbers MK282837 to 42.

**Table 1:** *Oesophagostomum* spp. *cox*1 reference sequences used in this study (n = 83).

**Species**	**Host**	**GenBank**	**Countries**	**Reference**
	**Species**	**accession number**		
*O. aculeatum*	*Macaca fuscata*	LC063900	Japan	Ota et al. (2015)
*O. aculeatum*	*Macaca fascicularis*	LC428762	Malaysia	Frias et al. (2019)
*O. aculeatum*	*Macaca fascicularis*	LC428765	Malaysia	Frias et al. (2019)
*O. aculeatum*	*Macaca fascicularis*	LC428773	Malaysia	Frias et al. (2019)
*O. aculeatum*	*Macaca fascicularis*	LC428776	Malaysia	Frias et al. (2019)
*O. aculeatum*	*Macaca fascicularis*	LC428777	Malaysia	Frias et al. (2019)
*O. aculeatum*	*Macaca fascicularis*	LC428778	Malaysia	Frias et al. (2019)
*O. aculeatum*	*Macaca fascicularis*	LC428779	Malaysia	Frias et al. (2019)
*O. aculeatum*	*Macaca fascicularis*	LC428781	Malaysia	Frias et al. (2019)
*O. aculeatum*	*Macaca fascicularis*	LC428782	Malaysia	Frias et al. (2019)
*O. aculeatum*	*Macaca fascicularis*	LC428783	Malaysia	Frias et al. (2019)
*O. aculeatum*	*Nasalis larvatus*	LC428786	Malaysia	Frias et al. (2019)
*O. aculeatum*	*Pongo pygmaeus*	LC428789	Malaysia	Frias et al. (2019)
*O. aculeatum*	*Pongo pygmaeus*	LC428791	Malaysia	Frias et al. (2019)
*O. aculeatum*	*Pongo pygmaeus*	LC428792	Malaysia	Frias et al. (2019)
*O. aculeatum*	*Pongo pygmaeus*	LC428793	Malaysia	Frias et al. (2019)
*O. aculeatum*	*Pongo pygmaeus*	LC428794	Malaysia	Frias et al. (2019)
*O. aculeatum*	*Pongo pygmaeus*	LC428796	Malaysia	Frias et al. (2019)
*O. aculeatum*	*Pongo pygmaeus*	LC428797	Malaysia	Frias et al. (2019)
*O. aculeatum*	*Pongo pygmaeus*	LC428798	Malaysia	Frias et al. (2019)
*O. aculeatum*	*Pongo pygmaeus*	LC428800	Malaysia	Frias et al. (2019)
*O. aculeatum*	*Pongo pygmaeus*	LC428801	Malaysia	Frias et al. (2019)
*O. aculeatum*	*Pongo pygmaeus*	LC428803	Malaysia	Frias et al. (2019)
*O. aculeatum*	*Pongo pygmaeus*	LC428807	Malaysia	Frias et al. (2019)
*O. aculeatum*	*Pongo pygmaeus*	LC428809	Malaysia	Frias et al. (2019)
*O. aculeatum*	*Pongo pygmaeus*	LC428811	Malaysia	Frias et al. (2019)
*O. aculeatum*	*Pongo pygmaeus*	LC428812	Malaysia	Frias et al. (2019)
*O. aculeatum*	*Pongo pygmaeus*	LC428813	Malaysia	Frias et al. (2019)
*O. aculeatum*	*Pongo pygmaeus*	LC428815	Malaysia	Frias et al. (2019)
*O. aculeatum*	*Pongo pygmaeus*	LC428816	Malaysia	Frias et al. (2019)
*O. aculeatum*	*Pongo pygmaeus*	LC428817	Malaysia	Frias et al. (2019)
*O. aculeatum*	*Pongo pygmaeus*	LC428818	Malaysia	Frias et al. (2019)
*O. aculeatum*	*Pongo pygmaeus*	LC428819	Malaysia	Frias et al. (2019)
*O. aculeatum*	*Pongo pygmaeus*	LC428820	Malaysia	Frias et al. (2019)
*O. aculeatum*	*Pongo pygmaeus*	LC428821	Malaysia	Frias et al. (2019)
*O. aculeatum*	*Pongo pygmaeus*	LC428822	Malaysia	Frias et al. (2019)
*O. aculeatum*	*Pongo pygmaeus*	LC428823	Malaysia	Frias et al. (2019)
*O. stephanostomum*	*Pan troglodytes schweinfurthii*	LC063867	Uganda	Ota et al. (2015)
*O. stephanostomum*	*Pan troglodytes schweinfurthii*	LC063868	Uganda	Ota et al. (2015)
*O. stephanostomum*	*Pan troglodytes schweinfurthii*	LC063871	Uganda	Ota et al. (2015)
*O. stephanostomum*	*Pan troglodytes schweinfurthii*	LC063876	Uganda	Ota et al. (2015)
*O. stephanostomum*	*Pan troglodytes schweinfurthii*	LC063877	Uganda	Ota et al. (2015)
*O. stephanostomum*	*Pan troglodytes schweinfurthii*	LC063879	Uganda	Ota et al. (2015)
*O. stephanostomum*	*Pan troglodytes schweinfurthii*	LC063881	Uganda	Ota et al. (2015)
*O. stephanostomum*	*Pan troglodytes schweinfurthii*	LC063882	Uganda	Ota et al. (2015)
*O. stephanostomum*	*Pan troglodytes schweinfurthii*	LC063883	Uganda	Ota et al. (2015)
*O. stephanostomum*	*Pan troglodytes schweinfurthii*	LC063886	Uganda	Ota et al. (2015)
*O. stephanostomum*	*Pan troglodytes schweinfurthii*	LC063887	Uganda	Ota et al. (2015)
*O. stephanostomum*	*Pan troglodytes schweinfurthii*	LC063888	Uganda	Ota et al. (2015)
*O. stephanostomum*	*Gorilla gorilla gorilla*	AB821032	Gabon	Makouloutou et al. (2014)
*O. stephanostomum*	*Gorilla gorilla gorilla*	AB821033	Gabon	Makouloutou et al. (2014)
*O. stephanostomum*	*Gorilla gorilla gorilla*	AB821034	Gabon	Makouloutou et al. (2014)
*O. stephanostomum*	*Gorilla gorilla gorilla*	AB821036	Gabon	Makouloutou et al. (2014)
*O. stephanostomum*	*Gorilla gorilla gorilla*	AB821037	Gabon	Makouloutou et al. (2014)
*O. stephanostomum*	*Gorilla gorilla gorilla*	AB821038	Gabon	Makouloutou et al. (2014)
*O. stephanostomum*	*Gorilla gorilla gorilla*	AB821039	Gabon	Makouloutou et al. (2014)
*O. stephanostomum*	*Gorilla gorilla gorilla*	AB821040	Gabon	Makouloutou et al. (2014)
*O. stephanostomum*	*Gorilla gorilla gorilla*	AB821041	Gabon	Makouloutou et al. (2014)
*O. stephanostomum*	*Gorilla gorilla gorilla*	AB821042	Gabon	Makouloutou et al. (2014)
*O. stephanostomum*	*Pan troglodytes*	AB821044	Gabon	Makouloutou et al. (2014)
*O. columbianum*	Sheep	KC715827	China	Zhao et al. (2013)
*O. columbianum*	Sheep	NC 023933	China	Zhao et al. (2013)
*Oesophagostomum* sp.	*Ovis aries*	MK282868	Brazil	Monteiro et al. (unpublished data)
*Oesophagostomum* sp.	*Ovis aries*	MK282869	Brazil	Monteiro et al. (unpublished data)
*Oesophagostomum* sp.	*Capra hircus*	MK282872	Brazil	Monteiro et al. (unpublished data)
*O. muntiacum*	*Muntiacus reevesi*	LC415114	Japan	Setsuda et al. (2020)
*O. asperum*	Goat	NC_023932	China	Zhao et al. (2013)
*O. dentatum*	Pig	FM161882	China	Lin et al. (2012b)
*O. quadrispinulatum*	Pig	FM161883	China	Lin et al. (2012b)
*O. bifurcum*	*Pan troglodytes schweinfurthii*	LC063862	Uganda	Ota et al. (2015)
*O. bifurcum*	*Pan troglodytes schweinfurthii*	LC063863	Uganda	Ota et al. (2015)
*O. bifurcum*	*Pan troglodytes schweinfurthii*	LC063864	Uganda	Ota et al. (2015)
*O. bifurcum*	*Pan troglodytes schweinfurthii*	LC063865	Uganda	Ota et al. (2015)
*O. bifurcum*	*Papio ursinus*	LC063889	South Africa	Ota et al. (2015)
*O. bifurcum*	*Papio ursinus*	LC063890	South Africa	Ota et al. (2015)
*O. bifurcum*	*Papio ursinus*	LC063891	South Africa	Ota et al. (2015)
*O. bifurcum*	*Papio cynocephalus*	LC063892	Tanzania	Ota et al. (2015)
*O. bifurcum*	*Papio cynocephalus*	LC063893	Tanzania	Ota et al. (2015)
*O. bifurcum*	*Papio cynocephalus*	LC063894	Tanzania	Ota et al. (2015)
*O. bifurcum*	*Papio cynocephalus*	LC063895	Tanzania	Ota et al. (2015)
*O. bifurcum*	*Papio cynocephalus*	LC063896	Tanzania	Ota et al. (2015)
*O. bifurcum*	*Papio cynocephalus*	LC063897	Tanzania	Ota et al. (2015)
*O. bifurcum*	*Papio cynocephalus*	LC063898	Tanzania	Ota et al. (2015)

* *Unpublished*

Phylogenetic inferences were made using the Molecular Evolutionary Genetics Analysis (MEGA) v.7.0.20 software (Kumar et al., 2016). The maximum likelihood (ML) method was applied and the Hasegawa-Kishino-Yano model (HKY) with gamma distribution (G) (Hasegawa et al., 1985) was selected using the Bayesian information criterion (BIC) in MEGA v.7.0.20 (Kumar et al., 2016). The clade stability of the *cox*1 sequence tag topologies was evaluated using 1,000 bootstrap replicates.

The relationships in the haplotype network were inferred through median joining using the Network v.10.2.0 and DnaSP v.6 software (Rozas et al., 2017). Free vector images were used in the figures of the phylogenetic tree and haplotype network, to represent the hosts (Flaticon, 2022; SVG SILH, 2022). The genetic diversity indexes of *Oesophagostomum* populations were calculated using the Arlequin v.5.2.2 software (Excofer & Lischer, 2010). The Fst fixation index was determined on all populations, using the Arlequin v.5.2.2 software to estimate the genetic differentiation among populations, with a significance of 1,000 permutations (Excofer & Lischer, 2010). This study was approved by the Ethics Committee for the Use of Animals (LW-21/13 [P-4/13.3]) of Instituto Oswaldo Cruz (Fiocruz).

## Results

Eighty-seven fecal samples from pigs were analyzed and the positivity rate for strongyliform eggs through microscopy was 81.6% (71/87). Six sequences (637 bp) were identified as belonging to the genus *Oesophagostomum*. From NSN, four samples were characterized as *O. quadrispinulatum* and one as *O. columbianum.* In TER, one sample was characterized as *O. dentatum*. Three *cox*1 sequences showed overlapping peaks, thus indicating the presence of more than one species or genotype. Cloning of the fragments enabled identification of the species *O. quadrispinulatum* and *O. dentatum*. Fecal samples positive for strongyliform eggs which were negative for *Oesophagostomum* in the molecular analysis allowed the identification of *Trichostrongylus* sp. (n=1) and *Metastrongylus* spp. (n=10) through *cox*1 sequencing. Therefore, no *Oesophagostomum* species previously characterized in humans was found in the studied swine populations.

**Figure 2:**
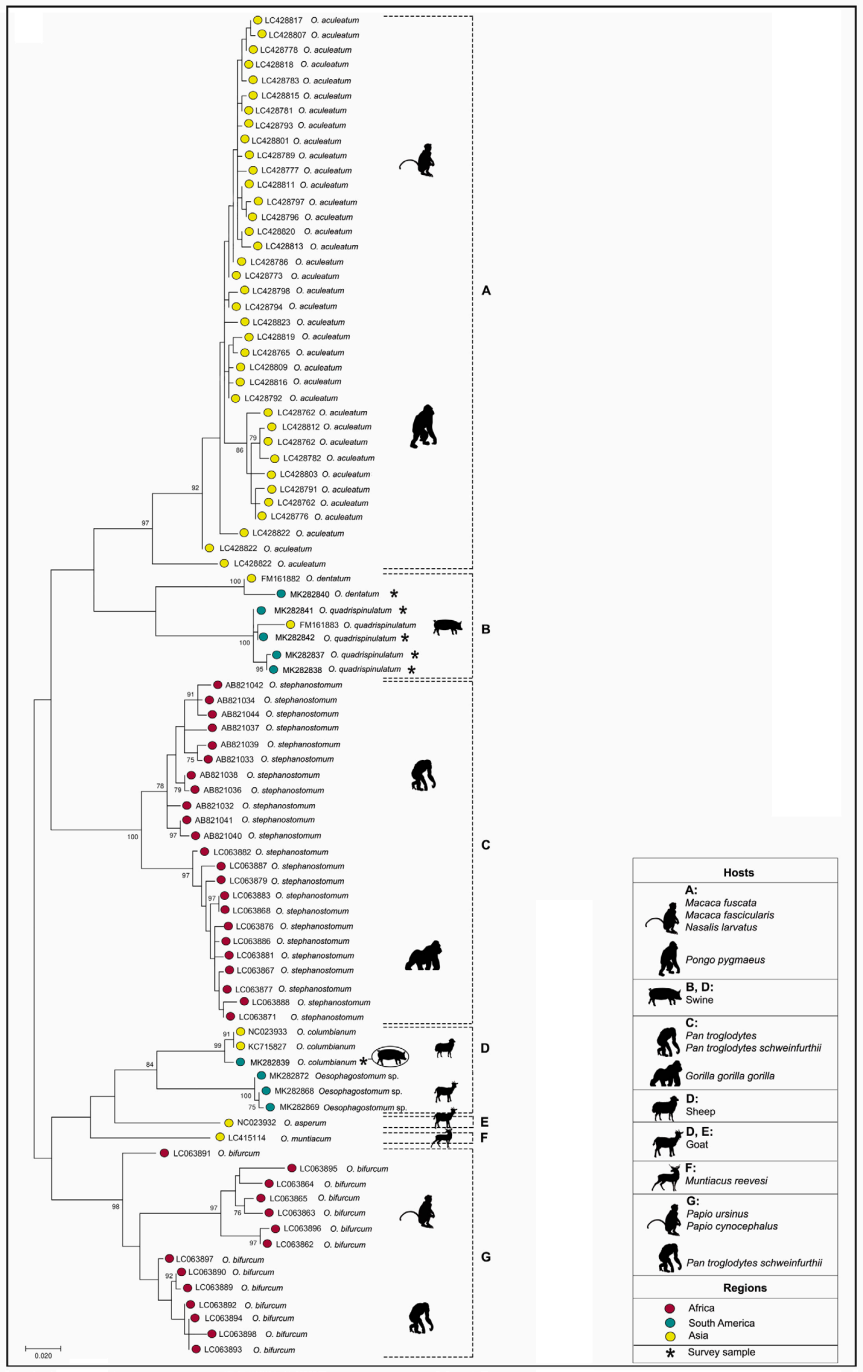
Maximum likelihood (ML) tree constructed using 401 bp *cox*1 sequences of *Oesophagostomum* spp.. Support for the branching order was determined by means of 1,000 bootstrap replicates, and only values > 70% were reported.

Alignment of the sequences of the present study in relation to 83 *Oesophagostomum* spp. reference sequences (401 bp *cox*1 sequences) from GenBank (Table 1) was performed. The ML phylogenetic tree (Figure 2) showed that the *Oesophagostomum* sequences were organized into three main groups. These groups included seven clades: i) clade A containing 37 sequences of *O. aculeatum* from Japan and Malaysia, identified in non-human primates (*Macaca fuscata, Macaca fascicularis, Nasalis larvatus* and *Pongo pygmaeus*); ii) clade B containing two sequences from *O. dentatum* from China and Brazil (TER) and five from *O. quadrispinulatum* from China and Brazil (NSN), all from pig hosts; iii) clade C containing 23 sequences of *O. stephanostomum* from Uganda and Gabon, detected in non-human primate hosts (*Pan troglodytes*, *Pan troglodytes schweinfurthii* and *Gorilla gorilla gorilla*); iv) clade D containing three sequences of *O. columbianum* from Asia and Brazil (NSN) identified in goats and one pig, and three of *Oesophagostomum* sp. from sheep and goats in Brazil; v) clade E containing a sequence of *O. asperum* from China, detected in a goat; vi) clade F containing a sequence of *O. muntiacum* from Japan, observed in a deer (*Muntiacus reevesi*); and vii) clade G containing 13 sequences of *O. bifurcum* from Uganda, Tanzania and South Africa, from non-human primate hosts (*Pan troglodytes schweinfurthii, Papio ursinus* and *Papio cynocephalus*).

In the study area, *O. quadrispinulatum* was the predominant species. The sequences of *O. quadrispinulatum* and *O. dentatum* were grouped in the same clade, in which there were only sequences from pigs (showing 99% similarity with the reference sequences), and were more closely related to *O. aculeatum* (cluster A). The *O. columbianum* sequence in the present study was grouped into the same clade as parasites obtained from small ruminants. Interestingly, three sequences that clustered close to *O. columbianum* (Figure 2) were located in another arm of the tree (showing 99% similarity with the reference sequence).

**Figure 3:**
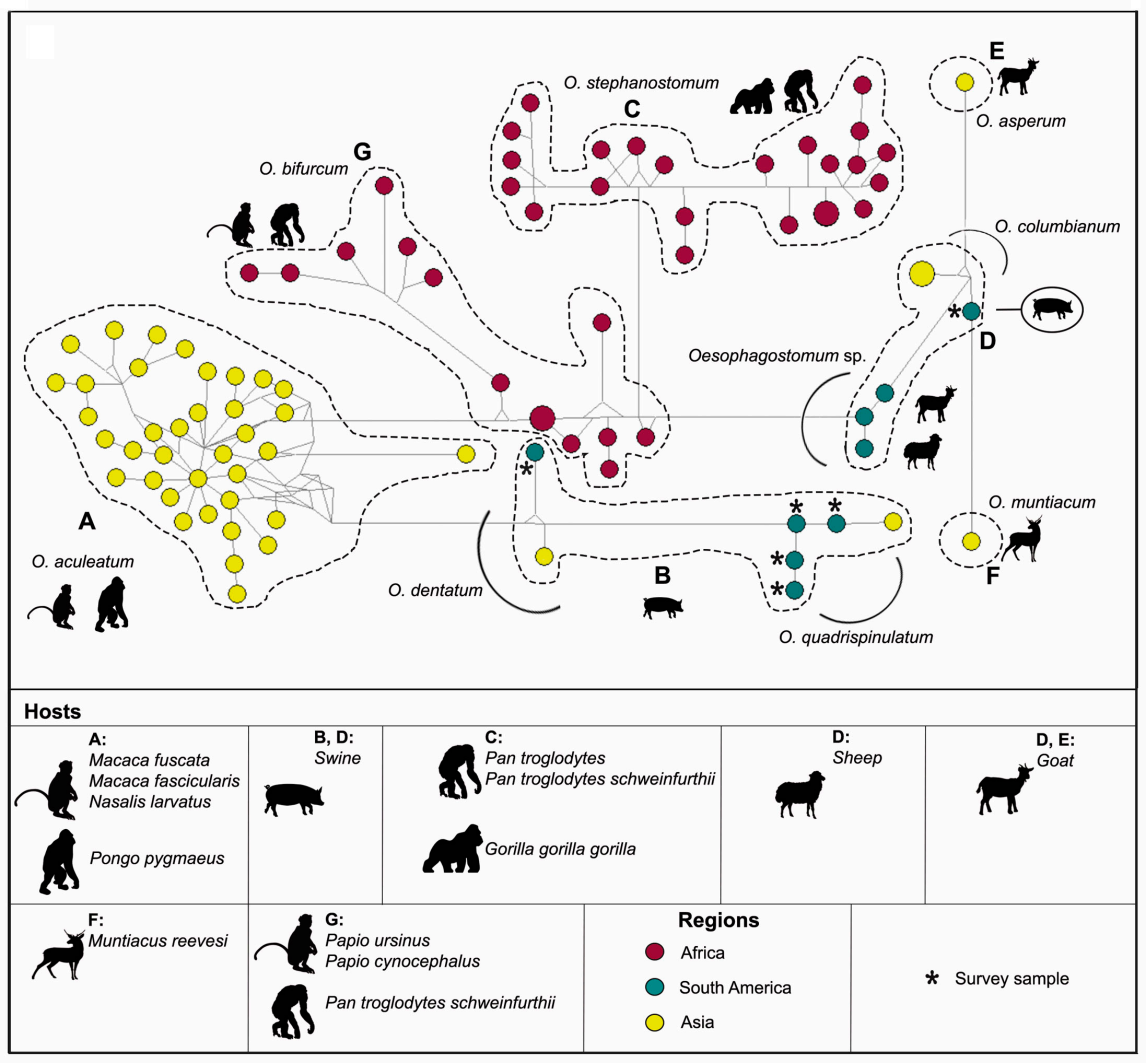
Maximum likelihood (ML) network of 401 bp *cox*1 locus of *Oesophagostomum* spp. The area of the circle was proportional to the sequence number.

The haplotype network (Figure 3) based on the *cox*1 locus showed topology similar to the phylogenetic tree. The 89 sequences used in the phylogenetic analyses were distributed into 86 haplotypes (Table 2). Six novel haplotypes of *Oesophagostomum* were identified in the present study. The species *O. aculeatum* showed a star shape, with a central haplotype, which has been identified in Asia. In general, the groups had long arms due to the number of polymorphisms identified among the species. Genetic diversity indices revealed high interspecific diversity in the genus *Oesophagostomum*, with H ± SD = 0.9992 ± 0.0018 and 129 polymorphic sites (Table 2). The intraspecific diversity varied according to the species. *Oesophagostomum columbianum* showed the lowest intraspecific variability with H ± SD = 0.6667 ± 0.3143 and 4 polymorphic sites. The genetic divergence (Fst) results were similar to the genetic diversity analyses (Table 3). The intraspecific divergence among specimens of *O. columbianum* was greater than the interspecific divergence of the samples analyzed (*O. columbianum* Asia Fst = 1; *O. bifurcum* Fst = 0.55). Furthermore, the Fst value between *O. quadrispinulatum* and *O. dentatum* was high: Fst = 1.

**Table 2:** Molecular diversity indexes of *Oesophagostomum* spp. based on *cox*1 locus (401 bp, n = 89).

Species (N)	Region (N)	Statistics					
		H ± SD	Nº of haplotypes	Nº of polymorphic sites	Nº of substitutions	Nº of transitions	Nº of transversions
*O. aculeatum* (37)	Asia (37)	1.0000 ± 0.0063	37	56	56	51	10
*O. bifurcum* (14)	Africa (14)	0.9890 ± 0.0314	13	54	54	47	9
*O. stephanostomum* (23)	Africa (23)	0.9960 ± 0.0142	22	51	54	48	6
*Oesophagostomum* sp. (3)	South America (3)	1.0000 ± 0.2722	3	2	2	2	0
O. columbianum** (3)	All (3)	0.6667 ± 0.3143	2	4	4	4	0
	Asia (2)	0.0000 ± 0.0000	1	0	0	0	0
*O. dentatum* ** (2)	All (2)	1.0000 ± 0.5000	2	7	7	6	1
O. quadrispinulatum** (5)	All (5)	1.0000 ± 0.1265	5	10	10	8	2
	**Brazil (4)**	1.0000 ± 0.1768	4	5	5	4	1
All* (89)		0.9992 ± 0.0018	86	129	129	99	63

H ± SD: gene diversity ± standard deviation;

All*: *O. aculeatum, O. asperum, O. bifurcum, O. muntiacum, O. stephanostomum, Oesophagostomum* sp., *O. columbianum, O. dentatum, O. quadrispinulatum.* Further details of reference sequences can be found in Table 1. In bold: sequences obtained in this study (Brazil);

** Groups formed only by one sequence have been removed.

**Table 3:** Population pairwise Fst values based on *cox*1 *Oesophagostomum* spp. (401 bp, n = 89).

**Population**	*O. aculeatum*_Asia	*O. asperum*_Asia	*O. bifurcum*_Africa	*O. muntiacum*_Asia	*O. stephanostomum*_Africa	*Oesophagostomum s*p._	*O. columbianum_*	*O. columbianum_*Asia	***O. columbianum*_Brazil**	*O. dentatum_*	*O. dentatum_*Asia	***O. dentatum*_Brazil**	*O. quadrispinulatum*_ Asia and Brazil	*O. quadrispinulatum_*Asia	***O. quadrispinulatum*_Brazil**	All*
						South America	Asia and Brazil			Asia and Brazil						
*O. aculeatum*_Asia																
*O. asperum*_Asia	0.83	0.00														
*O. bifurcum*_Africa	0.75	0.57	0.00													
*O. muntiacum*_Asia	0.84	1	0.60	0.00												
*O. stephanostomum*_Africa	0.78	0.65	0.64	0.68	0.00											
*Oesophagostomum* sp._South America	0.87	0.97	0.66	0.97	0.74	0.00										
*O. columbianum*_Asia and Brazil	0.86	0.94	0.64	0.94	0.69	0.95	0.00									
*O. columbianum_*Asia	0.86	1	0.63	1	0.68	0.98	-0.20	0.00								
***O. columbianum*_Brazil**	0.84	1	0.55	1	0.65	0.96	0.00	1	0.00							
*O. dentatum_*Asia and Brazil	0.83	0.86	0.67	0.87	0.69	0.94	0.92	0.93	0.86	0.00						
*O. dentatum_*Asia	0.82	1	0.61	1	0.65	0.97	0.94	1	1	-1	0.00					
***O. dentatum*_Brazil**	0.83	1	0.65	1	0.69	0.97	0.95	1	1	-1	1	0.00				
*O. quadrispinulatum*_ Asia and Brazil	0.85	0.90	0.70	0.90	0.73	0.94	0.92	0.92	0.90	0.88	0.88	0.89	0.00			
*O. quadrispinulatum_*Asia	0.84	1	0.60	1.00	0.69	0.98	0.95	1	1	0.84	1	1	0.20	0.00		
***O. quadrispinulatum*_Brazil**	0.85	0.94	0.69	0.94	0.72	0.96	0.94	0.95	0.94	0.91	0.93	0.93	-0.19	0.60	0.00	
All*	0.21	0.14	0.26	0.19	0.28	0.40	0.34	0.31	0.14	0.29	0.13	0.20	0.36	0.18	0.35	0.00

* *O. aculeatum, O. asperum, O. bifurcum, O. columbianum, O. dentatum, O. muntiacum, O. quadrispinulatum, O. stephanostomum, Oesophagostomum* sp.;

In bold: sequences obtained in this study (Brazil).

## Discussion

In the present study, the proportion of fecal samples from pigs that were positive for strongyliform eggs was higher than was found in Colombia (12.9%; 36/279) (Pinilla et al., 2020), India (19.9%; 74/371) (Yadav et al., 2021) and other region of Brazil, where it reached 46.6% (41/88) (Barbosa et al., 2015). Considering that the rearing systems reported by these authors were also extensive, our results may be explained by the handling and hygiene conditions of the pigs in the areas studied. Factors such as type of rearing, non-disinfection of drinking fountains and non-deworming are related to high frequencies of enteric helminths in pigs (Nansen & Roepstorff, 1999).

Making diagnoses based only on observation of eggs can lead to erroneous results due to similarities in morphology. Strongyliform eggs may belong to several species of pig parasites, including *Oesophagostomum* spp., hookworms, *Trichostrongylus* spp., *Hyostrongylus rubidus* and *Metastrongylus salmi*.

The present study used DNA barcoding to access species composition and genetic diversity. Primers for the mitochondrial target *cox*1 are “eclectic” due to high levels of intraspecific conservation and moderate interspecific variability, thereby providing identification of haplotypes and species in biological material (Hebert et al., 2004). It was possible to identify three distinct *Oesophagostomum* species in the area studied: *O. dentatum, O. quadrispinulatum* and *O. columbianum*. Interestingly, all sequences obtained in the present study were from different and undescribed haplotypes. None of these species are recognized as having zoonotic potential. Despite this, we cannot rule out the possibility of transmission of Strongylida parasites from pigs to human hosts. A previous study by our research group in NSN demonstrated that the strongyliform eggs found in human samples belong to the genus *Necator*, but not the species *N. americanus* (Monteiro et al., 2019).

Although *O. dentatum* and *O. quadrispinulatum* are parasites normally found in pigs, *O. columbianum* usually infects small ruminants. In the communities studied, pigs, goats and sheep are raised in close contact with each other, thus indicating the possibility of cross-host transmission. This type of management facilitates ingestion of goat and sheep feces by pigs, given that they are coprophagous, and enables passage of *O. columbianum* eggs or larvae through the digestive tract and presence of their DNA in fecal samples (pseudoparasitism), or even cross host transmission. In the present study, feces were collected fresh after defecation in the environment and, even though appropriate measures were taken at the time of collection, occurrence of contamination from the environment cannot be ruled out.

The phylogenetic tree and the haplotype network were structured based on mitochondrial DNA sequences and, therefore, were based on matrilineal inheritance. Within this perspective, *O. dentatum* and *O. quadrispinulatum* are closely related and formed a monophyletic group with two distinct clades. Similarly, phylogenetic analyses on *O. dentatum* and *O. quadrispinulatum* recovered from pigs in different regions of China generated a cluster with two clades that formed a monophyletic group (Lin et al., 2012a). The last authors used ribosomal DNA targets, which resulted in similar formation of a monophyletic group, with different groupings in the species *O. quadrispinulatum,* thereby indicating the presence of distinct genotypes or subspecies (Lin et al., 2014). Four distinct and novel haplotypes were identified in our *O. quadrispinulatum* sequences.

Pigs (*Sus scrofa domesticus*) are not autochthonous species from the Americas, having been introduced during the colonization process by Europeans. More recently, the introduction of the wild boar (*Sus scrofa scrofa*) in the 1990s led to their conversion into wild animals, as an exotic species, which population growing is uncontrolled in Brazil. Piauí is one of the states where its presence has not yet been registered. *Oesophagostomum dentatum* and *O. quadrispinulatum* infect domestic pigs in Brazil, Europe and Asia, with *O. dentatum* being identified in wild boar in Brazil as well (Silva & Müller, 2013; Li et al., 2017). It can be deduced that the process of introduction of pig farming in Brazil and its expansion also enabled the expansion of these helminth species.

Inclusion of sequences from other *Oesophagostomum* species in the phylogenetic analysis demonstrated the existence of seven distinct clades for this genus and that the *Oesophagostomum* species in pigs are closer to the non-human primate species *O. aculeatum* (which parasitizes monkeys and orangutans in Asia) and *O. stephanostomum* (which infects chimpanzees and gorillas in Africa). *Oesophagostomum columbianum* was found in pigs in the present study. A nucleotide BLAST (BLASTn) analysis in GenBank showed a similarity of 99% with *O. columbianum* in sheep from China (Zhao et al., 2013), and with *Oesophagostomum* sp. in goats and sheep from Brazil (Monteiro et al., unpublished data).

This is the first study exploring nucleotide sequences of *Oesophagostomum* in Brazil. These findings highlight the usefulness of molecular tools for investigating the taxonomy of strongyliform eggs observed in parasitological examinations, monitoring the presence of infection in herds ante-mortem, guiding control measures and providing data for studies on resistance to anthelmintics.
